# Antimicrobial susceptibility trends among gram-positive and -negative clinical isolates collected between 2005 and 2012 in Mexico: results from the Tigecycline Evaluation and Surveillance Trial

**DOI:** 10.1186/s12941-015-0116-y

**Published:** 2015-12-15

**Authors:** Rayo Morfin-Otero, Eduardo Rodriguez Noriega, Michael J. Dowzicky

**Affiliations:** Hospital Civil de Guadalajara, Fray Antonio Alcalde, Instituto de Patologia Infecciosa y Experimental, Centro Universitario Ciencias de la Salud, Universidad de Guadalajara, Guadalajara, Jalisco Mexico; Pfizer Inc, Collegeville, PA USA

**Keywords:** Tigecycline, Antimicrobial resistance, Mexico, Surveillance

## Abstract

**Background:**

The Tigecycline Evaluation and Surveillance Trial (T.E.S.T) is a global antimicrobial surveillance study of both gram-positive and gram-negative organisms. This report presents data on antimicrobial susceptibility among organisms collected in Mexico between 2005 and 2012 as part of T.E.S.T., and compares rates between 2005–2007 and 2008–2012.

**Method:**

Each center in Mexico submitted at least 200 isolates per collection year; including 65 gram-positive isolates and 135 gram-negative isolates. Minimum inhibitory concentrations (MICs) were determined using Clinical Laboratory Standards Institute (CLSI) broth microdilution methodology and antimicrobial susceptibility was established using the 2013 CLSI-approved breakpoints. For tigecycline US Food and Drug Administration (FDA) breakpoints were applied. Isolates of *E. coli* and *K. pneumoniae* with a MIC for ceftriaxone of >1 mg/L were screened for ESBL production using the phenotypic confirmatory disk test according to CLSI guidelines.

**Results:**

The rates of some key resistant phenotypes changed during this study: vancomycin resistance among *Enterococcus faecium* decreased from 28.6 % in 2005–2007 to 19.1 % in 2008–2012, while β-lactamase production among *Haemophilus influenzae* decreased from 37.6 to 18.9 %. Conversely, methicillin-resistant *Staphylococcus aureus* increased from 38.1 to 47.9 %, meropenem-resistant *Acinetobacter* spp. increased from 17.7 to 33.0 % and multidrug-resistant *Acinetobacter* spp. increased from 25.6 to 49.7 %. The prevalence of other resistant pathogens was stable over the study period, including extended-spectrum β-lactamase-positive *Escherichia coli* (39.0 %) and *Klebsiella pneumoniae* (25.0 %). The activity of tigecycline was maintained across the study years with MIC_90_s of ≤2 mg/L against *Enterococcus* spp., *S. aureus*, *Streptococcus agalactiae*, *Streptococcus pneumoniae*, *Enterobacter* spp., *E. coli*, *K. pneumoniae*, *Klebsiella oxytoca*, *Serratia marcescens*, *H. influenzae*, and *Acinetobacter* spp. All gram-positive organisms were susceptible to tigecycline and susceptibility among gram-negatives ranged from 95.0 % for *K. pneumoniae* to 99.7 % for *E. coli*.

**Conclusion:**

Antimicrobial resistance continues to be high in Mexico. Tigecycline was active against gram-positive and gram-negative organisms, including resistant phenotypes, collected during the study.

## Background

The widespread occurrence of antimicrobial resistance among bacterial pathogens is a global concern, and infections caused by resistant bacteria are now frequent events in hospitalized or community patients. Countries in Latin America are recognised to have high levels of resistance and antimicrobial susceptibility has decreased among many pathogens in Mexico in recent years [1–8]. For example, in one tertiary care hospital in Mexico susceptibility to meropenem among *Acinetobacter baumannii* decreased from 91.7 % in 1999 to 11.8 % in 2011 [8].

The Tigecycline Evaluation and Surveillance Trial (T.E.S.T.) is a global in vitro antimicrobial surveillance study which started in 2004 and began collecting gram-positive and gram-negative isolates from Mexican centers in 2005. Susceptibility is tested against a range of antimicrobials, including tigecycline, by each medical center before shipping to a central laboratory; the central laboratory then carries out data validation and the collation of the T.E.S.T. database. In this report, we compare data for isolates collected between 2005–2007 and 2008–2012 as well as presenting data for 2005–2012 as a whole. This report serves as an update to some of the data presented in Rossi et al. [9], who presented data on antimicrobial susceptibility across Latin America between 2004 and 2007. Data for *S. aureus* collected across Latin America between 2004 and 2010 was previously published by Garza-González and Dowzicky [10] and data on gram-negative organisms collected across Latin America between 2004 and 2010 was previously published by Fernández-Canigia and Dowzicky [11]. These reports also contain data from Mexican centers which are included in this analysis.

## Methods

In total, there were 16 centers in Mexico over the study period (1 center in 2005; 9 in 2006; 10 in 2007; 10 in 2008; 10 in 2009; 10 in 2010; 4 in 2011; and 15 in 2012). All centers did not participate in all years. The maximum number of years any one center participated for was 7 years. This was the case for two centers. One center participated for six years and four centers participated for 5 years. The remaining nine centers participated for between 2 and 4 years.

### Isolates collection

Each participating centre submitted at least 200 isolates per collection year; including 65 gram-positive isolates [*Enterococcus* spp. (*E. faecium* and *E. faecalis*; n = 15), *S. aureus* (n = 25), *Streptococcus agalactiae* (n = 10), and *S. pneumoniae* (n = 15)] and 135 gram-negative isolates [*Acinetobacter* spp. (n = 15), *Enterobacter* spp. (n = 25), *Escherichia coli* (n = 25), *Haemophilus influenzae* (n = 15), *Klebsiella* spp. (*K. oxytoca* and *K. pneumoniae*; n = 25), *Pseudomonas aeruginosa* (n = 20), and *Serratia* spp. (n = 10)].

All body sites were considered acceptable sources, although a maximum of 25 % of isolates could be urinary in origin. Inclusion of any isolate in the study was independent of patient medical history, previous antimicrobial use, age or gender. Only a single isolate was permitted from each patient. Ethics committee approval was not required as the study does not collect patient identifying information.

### Antimicrobial susceptibility testing

All participating medical centres were responsible for isolate identification and susceptibility testing. Minimum inhibitory concentrations (MICs) for all pathogens and each antimicrobial agent in the T.E.S.T. panel were determined using Clinical Laboratory Standards Institute (CLSI) broth microdilution methodology [12], and either MicroScan^®^ panels (Dade Microscan Inc., West Sacramento, CA, USA) or Sensititre^®^ plates (TREK Diagnostic Systems, East Grinstead, UK). The core T.E.S.T. antimicrobial panel included: amoxicillin-clavulanate, ampicillin, ceftriaxone, imipenem or meropenem, levofloxacin, minocycline, piperacillin-tazobactam and tigecycline. Imipenem was replaced by meropenem in 2006 due to imipenem stability issues, while MicroScan^®^ panels were replaced by Sensititre^®^ plates. Gram-positive pathogens were tested against the core antimicrobials plus linezolid, penicillin and vancomycin; gram-negative isolates were tested against the core panel as well as amikacin, cefepime and ceftazidime. The *S. pneumoniae* test panel was expanded in 2006 to include azithromycin, clarithromycin, erythromycin and clindamycin.

All isolates of *E. coli* and *K. pneumoniae* were tested for extended-spectrum β-lactamase (ESBL) production while all *H. influenzae* were tested for β-lactamase production. Isolates of *E. coli* and *K. pneumoniae* with a MIC for ceftriaxone of >1 mg/L were screened for ESBL production using the CLSI phenotypic confirmatory disk test according to CLSI guidelines [13] using cefotaxime (30 µg), cefotaxime/clavulanic acid (30/10 µg), ceftazidime (30 µg), and ceftazidime/clavulanic acid (30/10 µg) disks (Oxoid, Inc., Ogdensburg, NY, USA). Mueller–Hinton agar used in testing was manufactured by Remel, Inc. (Lenexa, KS, USA). An increase of >5 mm in the inhibition zone of the combination disk when compared to that of the cephalosporin disk alone demonstrated ESBL production. *H. influenzae* isolates were tested for β-lactamase production using local methodologies.

Quality control (QC) strains were tested on each day of isolate testing. The QC strains used were *E. coli* ATCC 25922, *H. influenzae* ATCC 49247 and ATCC 49766, *P. aeruginosa* ATCC 27853, *E. faecalis* ATCC 29212, *S. aureus* ATCC 29213, and *S. pneumoniae* ATCC 49619. MIC data were used only if the daily QC test results were within ranges considered acceptable by CLSI [13].

All isolates were sent to International Health Management Associates, Inc. (IHMA, Schaumberg, IL, USA). IHMA were responsible for the organisation of isolate collection and transport and the management of a centralized database. IHMA were also responsible for carrying out isolate identification QC checks, which were conducted on approximately 10–15 % of isolates.

Antimicrobial susceptibility was established using CLSI-approved breakpoints. The 2013 version was used for all isolates in this study [13]. Tigecycline breakpoints, as published by the US Food and Drug Administration (FDA), were used in this analysis [14]. FDA tigecycline breakpoints for *E. faecalis* (vancomycin-susceptible) were used for all *Enterococcus* isolates.

### Statistical analysis

Comparison of susceptibility between the 2005–2007 and 2008–2012 time periods were analysed using the Cochran–Mantel–Haenszel test with SAS (Version 8.2). Because of the large number of hypothesis tests, significance was determined at p < 0.01. The Fisher’s exact test was used to analyse changes in the percentage of resistant phenotypes between the two time periods, again using SAS (version 8.2). In this test significance was defined at p < 0.05.

## Results

Demographic and source data for the isolates in this study are presented in Table 1.

**Table 1:** Demographic and source data for clinically important gram-positive and gram-negative isolates collected in Mexico, T.E.S.T. 2005–2012

	Gram-positive (%) (n = 2207)		Gram-negative (%) (n = 4860)		Gram-positive + Gram-negative (%) (n = 7067)		
	2005–2007 (n = 721)	2008–2012 (n = 1486)	2005–2007 (n = 1459)	2008–2012 (n = 3401)	2005–2007 (n = 2180)	2008–2012 (n = 4887)	2005–2012
Age group (years)							
≤18	18.0	21.5	16.2	18.3	16.8	19.3	18.5
19–65	60.5	61.1	60.9	61.5	60.7	61.4	61.2
≥66	17.6	14.5	18.8	17.1	18.4	16.3	17.0
Gender							
Female	48.7	46.1	48.5	44.3	48.5	44.9	46.0
Male	51.0	52.0	51.4	52.7	51.3	52.5	52.1
Source							
Bodily fluids	23.7	25.8	18.8	18.3	20.5	20.6	20.5
Central nervous system	0.3	0.5	0.5	0.1	0.5	0.2	0.3
Cardio-vascular system	9.8	23.1	16.9	24.8	14.5	24.3	21.3
Gastro-intestinal	1.0	0.2	1.7	1.0	1.5	0.7	1.0
Genital/urinary	12.6	7.4	12.3	10.4	12.4	9.5	10.4
Head/ears/eyes/nose/throat	10.0	3.5	3.9	2.2	5.9	2.6	3.6
Integumentary	12.5	16.2	14.3	14.4	13.7	15.0	14.6
Instruments	3.2	3.3	4.2	6.1	3.9	5.3	4.9
Lymph	0.7	0.0	0.1	0.0	0.3	0.0	0.1
Reproductive	5.8	4.2	1.4	0.8	2.9	1.9	2.2
Respiratory	18.7	14.3	24.4	20.8	22.5	18.9	20.0
Skeletal	0.0	0.5	0.1	0.2	0.1	0.3	0.2
Muscular	1.5	0.3	0.9	0.2	1.1	0.2	0.5
Ward/clinic							
Clinic/office	6.7	6.5	3.5	5.4	4.5	5.7	5.4
Emergency room	3.3	4.7	4.5	4.0	4.1	4.2	4.2
Medicine general	30.9	40.2	31.6	41.3	31.4	40.9	38. 0
Medicine ICU	8.0	7.0	9.6	11.5	9.1	10.1	9.8
Nursing home/rehab	0.0	0.1	0.1	0.1	0.1	0.1	0.1
Pediatric general	4.0	6.5	4.3	5.3	4.2	5.7	5.2
Pediatric ICU	3.7	7.1	5.2	6.4	4.7	6.6	6.0
Surgery general	4.0	9.6	6.7	9.1	5.8	9.2	8.2
Surgery ICU	1.0	1.5	1.7	1.0	1.5	1.1	1.2
In/outpatient							
Inpatient	51.7	71.8	59.2	74.5	56.7	73.7	68.4
Outpatient	10.0	11.3	8.2	9.5	8.8	10.0	9.7

Table 2 presents data on the antimicrobial susceptibility of gram-positive and gram-negative isolates collected in Mexico between 2005 and 2012. Among gram-positive isolates ≥99 % were susceptible to linezolid, tigecycline and vancomycin. The one exception to this was *E. faecium* as only 75.0 % of isolates were susceptible to vancomycin. For *S. pneumoniae* susceptibility data for isolates from cerebrospinal fluid (CSF) are presented separately from data for isolates from other culture sources. Among non-CSF isolates there was a statistically significant decrease in susceptibility between the two time periods for amoxicillin-clavulanate and penicillin (p < 0.01). For isolates of *E faecalis* rates of susceptibility were similar between 2005–2007 and 2008–2012; however, for *E. faecium* rates of susceptibility were higher in 2008–2012 than in 2005–2007 for all antimicrobials with less than 100 % susceptibility and in the cases of ampicillin and penicillin these differences were statistically significant (p < 0.01).

**Table 2:** Antimicrobial susceptibility [MIC_90_ (mg/L), % susceptible] of clinically important gram-positive and gram-negative isolates collected in Mexico, T.E.S.T. 2005–2012

Pathogen	2005–2007		2008–2012		2005–2012	
	MIC_90_	%S	MIC_90_	%S	MIC_90_	%S
Gram-positive						
*E. faecalis*	n = 142		n = 332		n = 474	
Ampicillin	2	100	2	97.9	2	98.5
Levofloxacin	≥64	58.5	≥64	56.0	≥64	56.8
Linezolid	2	100	2	99.1	2	99.4
Minocycline	≥16	30.3	≥16	32.2	≥16	31.6
Penicillin	4	100	8	97.3	4	98.1
Tigecycline	0.25	100	0.25	100	0.25	100
Vancomycin	2	100	2	99.4	2	99.6
*E. faecium*	n = 42		n = 94		n = 136	
Ampicillin	≥32	19.0	≥32	53.2^a^	≥32	42.6
Levofloxacin	≥64	19.0	≥64	30.9	≥64	27.2
Linezolid	2	100	2	100	2	100
Minocycline	≥16	54.8	≥16	68.1	≥16	64.0
Penicillin	≥16	21.4	≥16	51.1^a^	≥16	41.9
Tigecycline	0.12	100	0.25	100	0.25	100
Vancomycin	≥64	66.7	≥64	78.7	≥64	75.0
*S. aureus*	n = 294		n = 728		n = 1022	
Levofloxacin	32	60.9	32	50.3^a^	32	53.3
Linezolid	2	100	2	100	2	100
Minocycline	0.5	99.7	1	98.2	1	98.6
Tigecycline	0.25	100	0.25	100	0.25	100
Vancomycin	1	100	1	100	1	100
*S. agalactiae*	n = 114 (33/81)		n = 173		n = 287 (33/254)	
Ampicillin	0.12	100	0.12	100	0.12	100
Ceftriaxone	0.12	100	0.12	100	0.12	100
Levofloxacin	1	99.1	1	98.8	1	99.0
Linezolid	1	100	1	100	1	100
Meropenem	≤0.12	100	0.25	100	≤0.12	100
Minocycline	≥16	23.7	≥16	21.4	≥16	22.3
Penicillin	0.12	100	0.12	100	0.12	100
Tigecycline	0.06	100	0.12	100	0.12	100
Vancomycin	0.5	100	1	100	0.5	100
*S. pneumoniae,* non-CSF	n = 120 (19/101) (78^b^)		n = 141 (122^b^)		n = 261 (19/242) (200^b^)	
Amoxicillin-clavulanate	1	99.2	4	86.5^a^	2	92.3
Azithromycin	64	69.2	≥128	54.9	≥128	60.5
Ceftriaxone	1	98.3	1	90.8	1	94.3
Clarithromycin	≥128	70.5	≥128	55.7	≥128	61.5
Clindamycin	≥128	84.6	≥128	78.7	≥128	81.0
Erythromycin	64	67.9	≥128	54.9	64	60.0
Imipenem	0.5	84.2	–	–	–	–
Levofloxacin	1	99.2	2	100	1	99.6
Linezolid	1	100	1	100	1	100
Meropenem	0.5	73.3	0.5	62.4	0.5	66.9
Minocycline	8	65.8	≥16	52.5	≥16	58.6
Penicillin^c^						
Oral	2	46.7	4	22.7^a^	2	33.7
Parenteral (non-meningitis)	2	97.5	4	89.4^a^	2	93.1
Parenteral (meningitis)	2	46.7	4	22.7^a^	2	33.7
Tigecycline	0.06	100	0.06	100	0.06	100
Vancomycin	0.5	100	0.5	100	0.5	100
*S. pneumoniae*, CSF	n = 9 (2/7) (8^b^)		n = 18 (0/18) (16^b^)		n = 27 (2/25) (24^b^)	
Azithromycin	–	[8]	≥128	75.0	32	83.3
Ceftriaxone	–	[8]	1	83.3	1	85.2
Clarithromycin	–	[8]	8	75.0	4	83.3
Clindamycin	–	[8]	64	87.5	0.25	91.7
Erythromycin	–	[8]	16	81.3	4	87.5
Imipenem	–	[2]	–	–	–	–
Levofloxacin	–	[9]	2	100	2	100
Linezolid	–	[9]	2	100	2	100
Meropenem	–	[6]	0.5	66.7	0.5	72.0
Minocycline	–	[6]	≥16	33.3	≥16	44.4
Penicillin^c^						
Parenteral (meningitis)	–	[5]	2	27.8	2	37.0
Tigecycline	–	[9]	0.06	100	0.06	100
Vancomycin	–	[9]	1	100	0.5	100
Gram-negative						
*Enterobacter* spp.	n = 283 (58/225) (277^d^)		n = 530		n = 813 (58/755) (807^d^)	
Amikacin	32	86.9	16	94.0^a^	16	91.5
Amoxicillin-clavulanate	≥64	7.1	≥64	11.9	≥64	10.2
Ampicillin	≥64	1.1	≥64	8.3^a^	≥64	5.8
Cefepime	16	87.3	16	89.4	16	88.7
Ceftriaxone	≥128	56.5	≥128	59.1	≥128	58.2
Imipenem	0.5	100	–	–	–	–
Levofloxacin	8	85.9	8	85.7	8	85.7
Meropenem	0.25	97.3	0.5	95.5	0.5	96.0
Minocycline	≥32	67.5	≥32	60.8	≥32	63.1
Piperacillin-tazobactam	128	76.7	128	76.8	128	76.8
Tigecycline	1	96.5	1	96.6	1	96.6
*E. coli*	n = 333 (99/234)		n = 863 (17/846)		n = 1196 (116/1080)	
Amikacin	8	96.1	16	91.4^a^	16	92.7
Amoxicillin-clavulanate	32	45.0	≥64	37.1^a^	32	39.3
Ampicillin	≥64	15.0	≥64	14.0	≥64	14.3
Cefepime	≥64	64.6	≥64	63.8	≥64	64.0
Ceftriaxone	≥128	45.3	≥128	43.5	≥128	44.0
Imipenem	0.5	99.0	≤0.06	100	0.25	99.1
Levofloxacin	≥16	35.7	≥16	33.8	≥16	34.4
Meropenem	0.25	95.3	0.25	98.6	0.25	97.9
Minocycline	16	64.3	≥32	49.1^a^	≥32	53.3
Piperacillin-tazobactam	16	90.1	128	74.3^a^	64	78.7
Tigecycline	0.5	99.7	0.5	99.7	0.5	99.7
*K. oxytoca*	n = 45 (10/35) (44^d^)		n = 91 (1/90)		n = 136 (11/125) (135^d^)	
Amikacin	8	97.8	8	94.5	8	95.6
Amoxicillin-clavulanate	32	84.4	32	68.1	32	73.5
Ampicillin	≥64	0.0	≥64	2.2	≥64	1.5
Cefepime	8	93.3	32	82.4	16	86.0
Ceftriaxone	32	75.6	≥128	68.1	≥128	70.6
Imipenem	0.25	100	–	[1]	0.25	100
Levofloxacin	≥16	84.4	≥16	73.6	≥16	77.2
Meropenem	0.25	100	0.5	95.6	0.25	96.8
Minocycline	16	75.6	16	74.7	16	75.0
Piperacillin-tazobactam	8	95.6	32	83.5	32	87.5
Tigecycline	1	97.8	1	97.8	1	97.8
*K. pneumoniae*	n = 236 (66/170) (233^d^)		n = 616		n = 852 (66/786) (849^d^)	
Amikacin	16	91.1	≥128	83.9^a^	64	85.9
Amoxicillin-clavulanate	32	67.8	≥64	55.5^a^	≥64	58.9
Ampicillin	≥64	1.3	≥64	2.8	≥64	2.4
Cefepime	4	93.6	≥64	80.5^a^	32	84.2
Ceftriaxone	64	68.2	≥128	56.2^a^	≥128	59.5
Imipenem	1	100	–	–	–	–
Levofloxacin	≥16	78.0	≥16	73.9	≥16	75.0
Meropenem	0.12	98.2	0.5	94.6	0.5	95.4
Minocycline	16	70.8	≥32	55.5^a^	≥32	59.7
Piperacillin-tazobactam	64	82.6	≥256	70.1^a^	≥256	73.6
Tigecycline	1	97.9	2	95.0	2	95.8
*S. marcescens*	n = 102 (26/76) (101^d^)		n = 211		n = 313 (26/287) (312^d^)	
Amikacin	64	77.5	32	86.3	64	83.4
Amoxicillin-clavulanate	≥64	15.7	≥64	7.6^a^	≥64	10.2
Ampicillin	≥64	5.0	≥64	4.7	≥64	4.8
Cefepime	16	87.3	8	90.0	16	89.1
Ceftriaxone	≥128	64.7	64	69.7	≥128	68.1
Imipenem	2	88.5	–	–	–	–
Levofloxacin	4	85.3	8	85.3	8	85.3
Meropenem	0.5	93.4	0.5	92.9	0.5	93.0
Minocycline	16	74.5	16	50.2^a^	16	58.1
Piperacillin-tazobactam	128	81.4	64	82.9	64	82.4
Tigecycline	2	97.1	2	95.3	2	95.8
*H. influenzae*	n = 117 (24/93)		n = 111 (5/106)		n = 228 (29/199)	
Amoxicillin-clavulanate	2	98.3	4	97.3	2	97.8
Ampicillin	32	59.8	32	79.3^a^	32	69.3
Cefepime	≤0.5	98.3	≤0.5	100	≤0.5	99.1
Ceftriaxone	0.12	100	0.12	97.3	0.12	98.7
Imipenem	0.5	100	–	[5]	0.5	100
Levofloxacin	0.03	100	0.03	100	0.03	100
Meropenem	0.25	100	0.12	100	0.25	100
Minocycline	1	100	1	98.2	1	99.1
Piperacillin-tazobactam	≤0.06	99.1	0.12	99.1	0.12	99.1
Tigecycline	0.25	99.1	0.25	97.3	0.25	98.2
*Acinetobacter* spp.	n = 129 (33/96)		n = 324		n = 453 (33/420)	
Amikacin	≥128	60.5	≥128	45.1^a^	≥128	49.4
Cefepime	32	56.6	≥64	51.2	≥64	52.8
Ceftazidime	≥64	28.7	≥64	30.6	≥64	30.0
Ceftriaxone	≥128	28.7	≥128	30.9	≥128	30.2
Imipenem	2	97.0	–	–	–	–
Levofloxacin	8	44.2	≥16	40.1^a^	≥16	41.3
Meropenem	16	76.0	≥32	63.3^a^	≥32	66.2
Minocycline	4	93.8	16	77.5^a^	16	82.1
Piperacillin-tazobactam	≥256	46.5	≥256	42.0	≥256	43.3
*P. aeruginosa*	n = 214 (70/144)		n = 655 (1/654)		n = 869 (71/798)	
Amikacin	64	71.5	≥128	64.3	≥128	66.1
Cefepime	≥64	62.6	≥64	59.5	≥64	60.3
Ceftazidime	≥64	51.9	≥64	50.5	≥64	50.9
Imipenem	≥32	54.3	–	[0]	≥32	53.5
Levofloxacin	≥16	59.8	≥16	58.3	≥16	58.7
Meropenem	≥32	56.3	≥32	56.0	≥32	56.0
Piperacillin-tazobactam	≥256	61.2	≥256	56.6	≥256	57.8

n values given in parentheses indicate the number of isolates tested against imipenem and meropenem, respectively. When no values are given in parenthesis, all isolates were tested against meropenem

When <10 isolates MIC_90_ data are not presented and the number of isolates susceptible or resistant are presented in parenthesis

When no isolates were tested against imipenem between 2008 and 2012, imipenem data for 2005–2012 are not presented as the only data available are for the 2005–2007 period

*CSF* cerebrospinal fluid

^a^Indicates a statistically significant change in susceptibility (p < 0.01 by the Cochran–Mantel–Haenszel test) between 2005–2007 and 2008–2012

^b^Against *S. pneumoniae* the n values given in parenthesis indicate the number of isolates tested against imipenem, meropenem or macrolides/clindamycin

^c^Against *S. pneumoniae* from non-cerebrospinal sources (non-CSF) three sets of breakpoints were applied: penicillin parenteral (non-meningitis); penicillin parenteral (meningitis), and penicillin oral. For isolates from CSF the penicillin parenteral (meningitis) breakpoints were applied

^d^Against Enterobacteriaceae the n value in parenthesis the number of isolates tested against ampicillin

The activity of the antimicrobial panel against the gram-negative organisms varied with susceptibility to the carbapenems and tigecycline at ≥95 % against *Enterobacter* spp., *E. coli*, *K. oxytoca* and *K. pneumoniae* when examining the 2005–2012 data. Susceptibility among *Acinetobacter* spp. and *P. aeruginosa* was lower. The MIC_90_ for tigecycline against *Acinetobacter* spp. was 2 mg/L for the 2005–2012 and 0.5 and 2 mg/L for the 2005–2007 and 2008–2012 time periods, respectively. Decreases in susceptibility among the *E. coli* submitted were noted for a number of antimicrobials with the largest decreases in susceptibility seen for minocycline and piperacillin-tazobactam. Both these decreases were considered statistically significant (p < 0.0001). For *K. pneumoniae* statistically significant (p < 0.01) decreases in susceptibility to minocycline, piperacillin-tazobactam, amoxicillin-clavulanate and ceftriaxone were seen between the two time periods. Susceptibility among *Acinetobacter* spp. was lower in 2008–2012 than in 2005–2007 for the majority of antimicrobial agents and decreases in susceptibility to amikacin, levofloxacin, meropenem, and minocycline were considered to be statistically significant (p < 0.01).

Antimicrobial susceptibility among MRSA, methicillin-susceptible *S. aureus*, ESBL-positive *E. coli* and *K. pneumoniae* and MDR *Acinetobacter* spp. are presented in Table 3. Greater than 97 % of *S. aureus* were susceptible to linezolid, minocycline, tigecycline and vancomycin irrespective of methicillin status. The carbapenems and tigecycline have the highest rates of susceptibility against ESBL-positive *E. coli* and *K. pneumoniae*. The MIC_90_ for tigecycline against MDR *Acinetobacter* spp. was 2 mg/L for the 2005–2012 time period; between 2005 and 2007 the MIC_90_ was 1 mg/L and between 2008 and 2012 was 4 mg/L.

**Table 3:** Antimicrobial susceptibility [MIC_90_ (mg/L), % susceptible, % resistant] of methicillin-resistant *S. aureus*, methicillin-susceptible *S. aureus*, extended-spectrum β-lactamase-positive *E. coli* and *K. pneumoniae* and multidrug-resistant *Acinetobacter* spp., collected in Mexico, T.E.S.T. 2005–2012

Pathogen	2005–2007			2008–2012			2005–2012		
	MIC_90_	%S	%R	MIC_90_	%S	%R	MIC_90_	%S	%R
*S. aureus*, MRSA	n = 112			n = 349			n = 461		
Levofloxacin	32	5.4	94.6	≥64	8.0	90.8	≥64	7.4	91.8
Linezolid	2	100	0.0	2	100	0.0	2	100	0.0
Minocycline	0.5	100	0.0	1	97.4	1.4	1	98.0	1.1
Tigecycline	0.25	100	–	0.5	100	–	0.5	100	–
Vancomycin	1	100	0.0	1	100	0.0	1	100	0.0
*S. aureus*, MSSA	n = 182			n = 379			n = 561		
Levofloxacin	0.5	95.1	3.3	2	89.2	7.9	1	91.1	6.4
Linezolid	4	100	0.0	4	100	0.0	4	100	0.0
Minocycline	1	99.5	0.5	0.5	98.9	0.8	0.5	99.1	0.7
Tigecycline	0.25	100	–	0.25	100	–	0.25	100	–
Vancomycin	1	100	0.0	1	100	0.0	1	100	0.0
*E. coli*, ESBL-positive	n = 134 (43/91)			n = 333 (9/324)			n = 467 (52/415)		
Amikacin	16	94.8	0.7	32	87.7	6.3^a^	32	89.7	4.7
Amoxicillin-clavulanate	32	26.1	29.1	32	16.8	40.2^a^	32	19.5	37.0
Ampicillin	≥64	0.7	99.3	≥64	2.1	97.6	≥64	1.7	98.1
Cefepime	≥64	23.9	58.2	≥64	26.1	60.7	≥64	25.5	60.0
Ceftriaxone	≥128	0.0	98.5	≥128	3.6	95.8	≥128	2.6	96.6
Imipenem	0.5	97.7	2.3	–	[9]	[0]	0.25	98.1	1.9
Levofloxacin	≥16	3.0	94.8	≥16	7.8	90.7	≥16	6.4	91.9
Meropenem	0.12	100	0.0	0.25	97.2	1.5	0.25	97.8	1.2
Minocycline	16	70.1	17.9	≥32	47.7	36.0^a^	≥32	54.2	30.8
Piperacillin-tazobactam	32	88.8	2.2	128	63.4	15.3^a^	128	70.7	11.6
Tigecycline	0.5	100	0.0	0.5	100	0.0	0.5	100	0.0
*K. pneumoniae*, ESBL-positive	n = 59 (15/44)			n = 154			n = 213 (15/198)		
Amikacin	≥128	76.3	16.9	≥128	59.1	32.5	≥128	63.8	28.2
Amoxicillin-clavulanate	≥64	25.4	40.7	≥64	13.6	53.9	≥64	16.9	50.2
Ampicillin	≥64	1.7	96.6	≥64	0.0	100	≥64	0.5	99.1
Cefepime	32	81.4	11.9	≥64	53.9	36.4^a^	≥64	61.5	29.6
Ceftriaxone	≥128	3.4	91.5	≥128	0.0	98.1	≥128	0.9	96.2
Imipenem	1	100	0.0	–	–	–	–	–	–
Levofloxacin	≥16	45.8	49.2	≥16	44.8	51.3	≥16	45.1	50.7
Meropenem	0.5	95.5	4.5	0.5	93.5	4.5	0.5	93.9	4.5
Minocycline	≥32	52.5	32.2	≥32	40.3	41.6	≥32	43.7	39.0
Piperacillin-tazobactam	≥256	49.2	25.4	≥256	37.0	37.0	≥256	40.4	33.8
Tigecycline	2	96.6	0.0	2	94.8	0.6	2	95.3	0.5
*Acinetobacter* spp., MDR	n = 33 (7/26)			n = 161			n = 194 (7/187)		
Amikacin	≥128	12.1	84.8	≥128	6.8	83.2	≥128	7.7	83.5
Cefepime	≥64	6.1	48.5	≥64	14.9	63.4	≥64	13.4	60.8
Ceftazidime	≥64	0.0	93.9	≥64	4.3	91.3	≥64	3.6	91.8
Ceftriaxone	≥128	0.0	100	≥128	1.9	96.3	≥128	1.5	96.9
Imipenem	–	[6]	[1]	–	–	–	–	–	–
Levofloxacin	≥16	0.0	90.9	≥16	1.2	95.7	≥16	1.0	94.8
Meropenem	16	34.6	53.8	≥32	29.8	64.6	≥32	30.5	63.1
Minocycline	2	90.9	6.1	≥32	62.1	28.6^a^	≥32	67.0	24.7
Piperacillin-tazobactam	≥256	9.1	84.8	≥256	3.1	92.5	≥256	4.1	91.2

n values given in parentheses indicate the number of isolates tested against imipenem and meropenem, respectively

When <10 isolates MIC_90_ data are not presented and the number of isolates susceptible or resistant are presented in parenthesis

When no isolates were tested against imipenem between 2008 and 2012, imipenem data for 2005–2012 are not presented as the only data available are for the 2005–2007 period

^a^A statistically significant change in susceptibility (p < 0.01 by the Cochran–Mantel–Haenszel test) between 2005–2007 and 2008–2012

A total of 504 carbapenem-resistant Enterobacteriaceae, *Acinetobacter* spp. and *P. aeruginosa* were identified in this study. Susceptibility data are presented in Table 4*K. oxytoca* were not included in this table as only 2 isolates were identified.

**Table 4:** Antimicrobial susceptibility [MIC_90_ (mg/L), % susceptible, % resistant] of carbapenem-resistant gram-negative organisms

Pathogen	2005–2007			2008–2012			2005–2012		
	MIC_90_	%S	%R	MIC_90_	%S	%R	MIC_90_	%S	%R
*Enterobacter* spp.	n = 5			n = 14			n = 19		
Amikacin	–	[0]	[3]	≥128	64.3	35.7	≥128	47.4	42.1
Amoxicillin-clavulanate	–	[0]	[5]	≥64	14.3	78.6	≥64	10.5	84.2
Ampicillin	–	[0]	[5]	≥64	14.3	85.7	≥64	10.5	89.5
Cefepime	–	[0]	[4]	≥64	50.0	35.7	≥64	36.8	47.4
Ceftriaxone	–	[0]	[5]	≥128	14.3	85.7	≥128	10.5	89.5
Levofloxacin	–	[0]	[5]	≥16	71.4	21.4	≥16	52.6	42.1
Minocycline	–	[5]	[0]	≥32	28.6	42.9	≥32	47.4	31.6
Piperacillin-tazobactam	–	[1]	[4]	≥256	42.9	42.9	≥256	36.8	52.6
Tigecycline	–	[5]	[0]	2	100	0.0	2	100	0.0
*E. coli*	n = 5			n = 8			n = 13		
Amikacin	–	[5]	[0]	–	[2]	[5]	≥128	53.8	38.5
Amoxicillin-clavulanate	–	[1]	[2]	–	[0]	[4]	≥64	7.7	46.2
Ampicillin	–	[3]	[2]	–	[0]	[8]	≥64	23.1	76.9
Cefepime	–	[3]	[1]	–	[1]	[7]	≥64	30.8	61.5
Ceftriaxone	–	[2]	[3]	–	[0]	[8]	≥128	15.4	84.6
Levofloxacin	–	[4]	[1]	–	[0]	[8]	≥16	30.8	69.2
Minocycline	–	[1]	[3]	–	[1]	[6]	≥32	15.4	69.2
Piperacillin-tazobactam	–	[2]	[1]	–	[4]	[4]	≥256	46.2	38.5
Tigecycline	–	[5]	[0]	–	[8]	[0]	2	100	0.0
*K. pneumoniae*	n = 3			n = 25			n = 28		
Amikacin	–	[1]	[0]	≥128	20.0	68.0	≥128	21.4	60.7
Amoxicillin-clavulanate	–	[0]	[3]	≥64	4.0	92.0	≥64	3.6	92.9
Ampicillin	–	[0]	[3]	≥64	0.0	100	≥64	0.0	100
Cefepime	–	[1]	[2]	≥64	12.0	76.0	≥64	14.3	75.0
Ceftriaxone	–	[0]	[3]	≥128	0.0	100	≥128	0.0	100
Levofloxacin	–	[0]	[2]	≥16	4.0	88.0	≥16	3.6	85.7
Minocycline	–	[2]	[0]	≥32	16.0	72.0	≥32	21.4	64.3
Piperacillin-tazobactam	–	[0]	[2]	≥256	8.0	80.0	≥256	7.1	78.6
Tigecycline	–	[3]	[0]	4	84.0	0.0	4	85.7	0.0
*S. marcescens*	n = 5			n = 13			n = 18		
Amikacin	–	[2]	[3]	≥128	38.5	53.8	≥128	38.9	55.6
Amoxicillin-clavulanate	–	[0]	[5]	≥64	0.0	100	≥64	0.0	100
Ampicillin	–	[0]	[5]	≥64	0.0	92.3	≥64	0.0	94.4
Cefepime	–	[2]	[2]	≥64	38.5	46.2	≥64	38.9	44.4
Ceftriaxone	–	[0]	[5]	≥128	23.1	76.9	≥128	16.7	83.3
Levofloxacin	–	[1]	[3]	≥16	23.1	69.2	≥16	22.2	66.7
Minocycline	–	[3]	[1]	≥32	7.7	76.9	≥32	22.2	61.1
Piperacillin-tazobactam	–	[0]	[5]	≥256	23.1	61.5	≥256	16.7	72.2
Tigecycline	–	[4]	[0]	8	84.6	15.4	8	83.3	11.1
*Acinetobacter* spp.	n = 18			n = 107			n = 125		
Amikacin	≥128	22.2	61.1	≥128	8.4	76.6	≥128	10.4	74.4
Cefepime	≥64	11.1	55.6	≥64	9.3	70.1	≥64	9.6	68.0
Ceftazidime	≥64	0.0	94.4	≥64	1.9	95.3	≥64	1.6	95.2
Ceftriaxone	≥128	0.0	100	≥128	0.9	99.1	≥128	0.8	99.2
Levofloxacin	≥16	0.0	66.7	≥16	2.8	90.7	≥16	2.4	87.2
Minocycline	2	94.4	5.6	≥32	64.5	28.0	≥32	68.8	24.8
Piperacillin-tazobactam	≥256	5.6	94.4	≥256	0.9	97.2	≥256	1.6	96.8
*P. aeruginosa*	n = 75			n = 226			n = 301		
Amikacin	64	38.7	42.7	≥128	28.8	55.3	≥128	31.2	52.2
Cefepime	≥64	25.3	60.0	≥64	21.2	61.1	≥64	22.3	60.8
Ceftazidime	≥64	25.3	64.0	≥64	15.9	76.5	≥64	18.3	73.4
Levofloxacin	≥16	26.7	69.3	≥16	19.5	70.8	≥16	21.3	70.4
Piperacillin-tazobactam	≥256	24.0	44.0	≥256	24.8	49.1	≥256	24.6	47.8

When <10 isolates MIC_90_ data are not presented and the number of isolates susceptible or resistant are presented in parenthesis

### Resistant phenotypes

Rates of resistant phenotypes for the three time periods are presented in Table 5. Rates of MRSA, meropenem-resistant *Acinetobacter* spp. and MDR *Acinetobacter* spp. increased between 2005–2007 and 2008–2012 with the rates of meropenem-resistant and MDR *Acinetobacter* spp. increasing significantly (p < 0.05). In comparison, β-lactamase production among *H. influenzae* decreased significantly (p < 0.05) between the two time periods.

**Table 5:** Rates of resistant phenotypes collected in Mexico, T.E.S.T. 2005–2012

Pathogen	2005–2007		2008–2012		2005–2012	
	N	n (%)	N	n (%)	N	n (%)
Gram-positive						
*E. faecalis*, vancomycin-R	142	0 (0.0)	332	2 (0.6)	474	2 (0.4)
*E. faecium*, vancomycin-R	42	12 (28.6)	94	18 (19.1)	136	30 (22.1)
*S. aureus*, methicillin-R	294	112 (38.1)	728	349 (47.9)	1022	461 (45.1)
Gram-negative						
*E. coli*, ESBL-positive	333	134 (40.2)	863	333 (38.6)	1196	467 (39.0)
*K. oxytoca*, ESBL-positive	45	7 (15.6)	91	14 (15.4)	136	21 (15.4)
*K. pneumoniae*, ESBL-positive	236	59 (25.0)	616	154 (25.0)	852	213 (25.0)
*H. influenzae*, BL-positive	117	44 (37.6)	111	21 (18.9)^a^	228	65 (28.5)
*Acinetobacter* spp., meropenem-R	96^b^	17 (17.7)	324	107 (33.0)^a^	453	124 (27.4)
*Acinetobacter* spp., MDR	129	33 (25.6)	324	161 (49.7)^a^	453	194 (42.8)

*BL* β-lactamase, *ESBL* extended-spectrum β-lactamase, *R* resistant, *MDR* multidrug resistant

^a^A statistically significant change in the percentage of resistant phenotype (p < 0.05 by the Fisher’s exact test) between 2005–2007 and 2008–2012

^b^A total of 129 *Acinetobacter* spp. were collected between 2005 and 2007; however, only 96 were tested against meropenem

## Discussion

Rates of antimicrobial resistance among both gram-positive and gram-negative organisms were high in this report from Mexico. Antimicrobial resistance is a recognized problem in Latin America with high levels of resistance among both gram-positive and gram-negative organisms [1–3]. There is a known relationship between antimicrobial use and resistance [15], and it can be surmised that one of the factors contributing to the high rates of resistance in Mexico is overuse of antimicrobials. Data on previous antimicrobial use is not collected by T.E.S.T.; however, such data would be of interest if the T.E.S.T. program were to develop in the future.

Overall, 45 % of *S. aureus* reported as MRSA, 22 % of *E. faecium* reported as vancomycin-resistant, 25 % of *K. pneumoniae* and 39 % of *E. coli* reported as ESBL-producers, 27 % of *Acinetobacter* spp. reported as resistant to meropenem and 42.8 % of *Acinetobacter* spp. were MDR. These results are similar to those presented by Jones et al. [3] for isolates collected in Mexico in 2011 where the MRSA rate was 48 % and 26 % of enterococci were vancomycin-resistant. ESBL rates among *E. coli* and *K. pneumoniae* were higher in the Jones et al. [3] report (71 and 56 %); however, as with this study, the rate of ESBLs was higher among *E. coli* than *K. pneumoniae*. SENTRY, which began in 1997, is also an antimicrobial surveillance program which collects isolates and antimicrobial susceptibility data from around the globe. Comparing susceptibility results for *E. coli* collected in Mexico through the SENTRY surveillance study (2008–2010) with results from this T.E.S.T. study show they were broadly comparable, with high levels of quinolone resistance occurring in both studies (34.4 % levofloxacin resistance in the current study, 35.4 % ciprofloxacin resistance in SENTRY) [16]. In addition, *Klebsiella* spp. susceptibility to piperacillin-tazobactam, ceftriaxone and cefepime was approximately 7–9 % higher in the SENTRY study than in T.E.S.T. for *K. pneumoniae*, although susceptibility was similar among other antimicrobial agents [16]. Carbapenem resistance among *K. pneumoniae* and *E. coli* was low in T.E.S.T. as has been previously reported for Mexico [17].

There was a variation in the susceptibility of *S. pneumoniae* to penicillin which was dependent on the breakpoints applied. For the 2005–2012 time period among non-CSF isolates susceptibility was 33.7 % using the oral or parenteral (meningitis) breakpoints but 93.1 % when using the parenteral (non-meningitis) breakpoints. Penicillin oral breakpoints are S ≤ 0.06 mg/L; I 0.12–1 mg/L; R ≥ 2 mg/L; whereas those for parenteral administered penicillin are: non-meningitis (S ≤ 2 mg/L; I 4 mg/L; R ≥ 8 mg/L) and meningitis (S ≤ 0.06 mg/L; R ≥ 0.12 mg/L). This highlights the importance of using the correct breakpoints when interpreting the susceptibility of an organism.

When comparing rates of resistance in Mexico with other countries the rate of MRSA (45 %) was similar to that reported for other Latin American countries such as Guatemala (49 %) and Panama (47 %) [3]. With limited treatment options, concern continues about the prevalence of MRSA globally and although rates have been reported to be decreasing in some regions, most notably North America and Europe [18, 19], the prevalence in other areas, particularly developing countries, is of increasing concern [20]. In their study of isolates collected between 2010 and 2014 Conceição et al. reported rates of 61.6 % in Angola, 25.5 % in São Tomé and Príncipe, 5.6 % in Cape Verde and 0.0 % in East Timor [21]. At 22 % the rate of vancomycin-resistant *E. faecium* was similar to that reported for enterococci in Brazil by Jones et al. (27 %) for 2011 [3]. It is also comparable to the rate of 18.5 % reported for Saudi Arabia in 2009–2010 [22]. The rates of ESBL producing *E. coli* and *K. pneumoniae*, although high, were relatively low when compared to some other countries. Sharma et al. reported that 67 % of *Klebsiella* spp. and 57 % of *E. coli* were ESBL producers in Jaipur, India in 2011–2012 [23]. Results for amikacin were similar in this study to the results reported for *E. coli* collected in Egypt but for *K. pneumoniae* susceptibility was higher in T.E.S.T. [24].

Antimicrobial susceptibility among *P. aeruginosa* was similar between the two time periods in this study and susceptibility rates were similar to those reported by Jones et al. for Latin America in 2011 [3]. In contrast, rates of susceptibility to amikacin and meropenem were lower than those reported by Gad et al. for *P. aeruginosa* isolates collected from three Egyptian hospitals [25]. In the case of *Acinetobacter* spp., resistance to meropenem increased from 17.7 % in 2005–2007 to 33.0 % in 2008–2012 in this T.E.S.T. program. Carbapenem resistance among *Acinetobacter* spp. has been reported both in Latin America and globally. For example, Oliveira et al. reported an increase in carbapenem resistance in Brazil from 7.4 % to 57.5 % between 1999 and 2008 and Aydin et al. reported an increase in meropenem resistance among *Acinetobacter* spp. collected from an ICU in Turkey from 26 % in 2008 to 95 % in 2011 [26, 27]. In addition a rate of 26 % was reported for a single center in India in 2013 although this was a decrease from the 33 % previously reported [28]. Other countries, such as Libya, are also reporting the emergence of carbapenem resistant *A. baumannii* [29]. These results demonstrate the variability in antimicrobial resistance between countries and with increasing globalization the importance of a global strategy to control the spread of resistant organisms.

Rossi et al. [9] examined the in vitro activity of tigecycline and comparator agents against gram-positive and gram-negative isolates from Latin America, including Mexico, between 2004 and 2007 as a part of the T.E.S.T. study. These data from Mexico are included in the current report but are updated with additional isolates. The most dramatic changes in susceptibility between 2005–2007 and 2008–2012 occurred among *S. pneumoniae*, *E. faecium*, *K. pneumoniae* and *K. oxytoca*: ≥10 % changes were observed for seven antimicrobial agents against non-CSF *S. pneumoniae* [amoxicillin-clavulanate, azithromycin, clarithromycin, erythromycin, meropenem, minocycline and penicillin (using oral or parenteral meningitis breakpoints], five antimicrobials against *E. faecium* (ampicillin, levofloxacin, minocycline, penicillin and vancomycin), five agents against *K. pneumoniae* (amoxicillin-clavulanate, cefepime, ceftriaxone, minocycline and piperacillin-tazobactam) and four antimicrobials against *K. oxytoca* (amoxicillin-clavulanate, cefepime, levofloxacin and piperacillin-tazobactam). All changes for *E. faecium* were increases in susceptibility, while for *S. pneumoniae*, *K. pneumoniae* and *K. oxytoca* decreases in susceptibility were seen. These changes in antimicrobial susceptibility may be due to a number of factors. Firstly, between the two time periods there was an increase in the number of isolates coming from inpatients in this study. As isolates from inpatients and outpatients are known to have different susceptibility profiles this could impact the susceptibility profile of the isolates as a whole. Also, increases in susceptibility can be due to improved antimicrobial stewardship whereas decreases in susceptibility may occur due to failures in stewardship and center specific outbreaks. Over the counter dispensing of antimicrobials is common in Latin America and in 2010 Mexico sought to enforce existing laws to reduce their consumption. This policy has been shown to have decreased consumption [30], although a trend for decreasing consumption had already been detected [31]. The relationship between antimicrobial consumption and resistance is well known.

Linezolid, meropenem, tigecycline and vancomycin retained their good in vitro activity against most T.E.S.T. pathogens between 2005–2007 and 2008–2012.

The in vitro activity for tigecycline reported here is also comparable with the literature. Gales et al. [32] reported that all isolates of *Enterococcus* spp., *S. aureus*, *S. pneumoniae*, and *H. influenzae* collected in Latin America between 2000 and 2002 were susceptible to tigecycline at MICs of ≤4 mg/L and MIC_90_s were ≤0.5 mg/L. Tigecycline retained this level of activity in the current study, with MIC_90_s for these organisms at ≤0.25 mg/L and 100 % tigecycline susceptibility reported among isolates of *Enterococcus* spp., *S. aureus* and *S. pneumoniae* and 98.2 % susceptibility reported among *H. influenzae* isolates. It is however, important to note that the breakpoints applied in this study are lower than the MIC cutoff used by Gales et al. [32]. The in vitro activity of tigecycline reported in this study for Mexico is also similar to that reported by Jones et al. [3] for gram-positive and gram-negative isolates collected across Latin America in 2011.

Breakpoints are not currently available for tigecycline against *Acinetobacter* spp. In this study the MIC_90_ for tigecycline was 0.5 mg/L between 2005–2007 and 2 mg/L for 2008–2012 and against MDR *Acinetobacter* spp. were one doubling dilution higher (1 and 4 mg/L, respectively). From the literature Garza-González et al. [4] reported an MIC_90_ for tigecycline of 0.5 mg/L among 550 *A. baumannii* isolates collected between 2006 and 2009 from a tertiary care teaching hospital in Mexico and Mendes et al. [33] reported a tigecycline MIC_90_ of 1 mg/L among 277 *Acinetobacter* spp. isolates from Mexico collected between 2005 and 2009.

As discussed above, rates of ESBL production are high in Mexico. In the current study, all ESBL-positive *E. coli* isolates and 95.3 % of ESBL-positive *K. pneumoniae* isolates were susceptible to tigecycline (data not shown). *E. cloacae* and *S. marcescens* are not examined for ESBLs as part of the T.E.S.T. study, but low levels of tigecycline non-susceptibility were observed in this study for both *Enterobacter* spp. (3.4 %) and *S. marcescens* (4.2 %). Silva-Sanchez et al. [34] have also reported good in vitro activity for tigecycline against ESBL-positive Enterobacteriaceae in Mexico (as well as MRSA), with >94 % of 1055 isolates reported as tigecycline susceptible. Tigecycline thus appears to be a potential treatment option in Mexico, where the prevalence of pathogens resistant to commonly-used antimicrobials is high.

Limitations of this study include the center repetition between years, with nine of the 16 centers participating for between two and four of the 8 years of study. The types of centers involved in surveillance studies can also influence results as large university hospitals and smaller community based hospitals can have differing levels of resistance. Both university and community based hospitals submitted isolates to the T.E.S.T. program in Mexico.

Surveillance studies such as SENTRY and T.E.S.T. are critical tools for monitoring the development and spread of resistance among important clinical pathogens, assisting healthcare professionals in making appropriate judgments for the best use of antimicrobials on regional or national levels and supporting antibiotic stewardship efforts [35, 36]. Tigecycline demonstrates good in vitro activity against most of the pathogens examined in this study, and should continue to be a useful option in the treatment of infectious diseases in Mexico.
